# Ketogenic Diet Impairs FGF21 Signaling and Promotes Differential Inflammatory Responses in the Liver and White Adipose Tissue

**DOI:** 10.1371/journal.pone.0126364

**Published:** 2015-05-14

**Authors:** Mohamed Asrih, Jordi Altirriba, Françoise Rohner-Jeanrenaud, François R. Jornayvaz

**Affiliations:** Division of Endocrinology, Diabetes, Hypertension and Nutrition, Geneva University Hospital, Rue Gabrielle-Perret-Gentil 4, 1211, Geneva, 14, Switzerland; Johns Hopkins University School of Medicine, UNITED STATES

## Abstract

**Background/Hypothesis:**

Beside its beneficial effects on weight loss, ketogenic diet (KD) causes dyslipidemia, a pro-inflammatory state involved in the development of hepatic steatosis, glucose intolerance and insulin resistance, although the latter is still being debated. Additionally, KD is known to increase fibroblast growth factor 21 (FGF21) plasma levels. However, FGF21 cannot initiate its beneficial actions on metabolism in these conditions. We therefore hypothesized and tested in the present study that KD may impair FGF21 signaling.

**Methods/Results:**

Using indirect calorimetry, we found that KD-fed mice exhibited higher energy expenditure than regular chow (RC)-fed mice associated with increased *Ucp1* levels in white adipose tissue (WAT), along with increased plasma FGF21 levels. We then assessed the effect of KD on FGF21 signaling in both the liver and WAT. We found that *Fgfr4* and *Klb* (β-klotho) were downregulated in the liver, while *Fgfr1* was downregulated in WAT of KD-fed mice. Because inflammation could be one of the mechanisms linking KD to impaired FGF21 signaling, we measured the expression levels of inflammatory markers and macrophage accumulation in WAT and liver and found an increased inflammation and macrophage accumulation in the liver, but surprisingly, a reduction of inflammation in WAT.We also showed that KD enhances lipid accumulation in the liver, which may explain hepatic inflammation and impaired *Fgfr4* and *Klb* expression. In contrast, import of lipids from the circulation was significantly reduced in WAT of KD-fed mice, as suggested by a downregulation of *Lpl* and *Cd36*. This was further associated with reduced inflammation in WAT.

**Conclusion:**

Altogether, these results indicate that KD could be beneficial for a given tissue but deleterious for another.

## Introduction

Obesity is the fastest-growing health problem worldwide. Obesity significantly increases the risk for major diseases such as type 2 diabetes and nonalcoholic fatty liver disease (NAFLD) [1]. Although the cellular mechanisms that link obesity to these diseases remain unknown, ectopic accumulation of lipids has been suggested as one of the causes [2, 3]. Indeed, in obese patients, lipids not only accumulate in white adipose tissue (WAT), but pathologically deposit in other organs such as liver and skeletal muscle [1, 4]. This leads to the development of insulin resistance, which represents an important risk factor for the development of type 2 diabetes and NAFLD [5].

To date, there are no reliable and efficient pharmacological approaches to treat NAFLD. Moreover, despite numerous drugs available for type 2 diabetes, the long term management of this disease remains disappointing. Nonetheless, it is well established that modest weight loss ameliorates insulin resistance. In this regard, ketogenic diet (KD) has been used as an efficient approach to achieve weight loss [6, 7]. Despite its beneficial effect on weight loss [8], KD induces adverse side-effects such as osteoporosis and hyperlipidemia on the long term [9, 10]. Moreover, KD has been reported to promote insulin resistance in humans [11], which may discourage the use of such diet in obese patients [12]. Several studies in animals have also shown that KD promotes hepatic steatosis, insulin resistance and glucose intolerance [13–15], although this has been questioned [16].

Interestingly, in mice, five weeks of KD feeding induces hepatic insulin resistance, while it does not impact insulin responsiveness in WAT [15]. However, another study indicates that 6 days of feeding using a different KD than the latter study impairs insulin sensitivity in wild-type mice due to insulin resistance in white adipose tissue, as shown by decreased Akt phosphorylation in this tissue [17]. Therefore, this suggests that there may be a differential response between WAT and liver to KD, or a differential response between different WAT depots. Further, KD was found to promote inflammation and macrophage accumulation in the liver, which is associated with hepatic insulin resistance [14]. Because the inflammatory state of WAT was not investigated in these studies, we aimed at determining whether KD modulates inflammation in WAT, which may explain the differential responses of WAT and liver to insulin.

Although the full spectrum of metabolic effects induced by KD is not completely characterized, it is suggested that fibroblast growth factor 21 (FGF21) is a major factor mediating part of the effects of this diet [18]. FGF21 is predominantly produced in the liver and mainly acts on adipocytes [19–22]. Recently, it has been shown that, by targeting adipose tissue, FGF21 promotes adiponectin production and secretion, which thereby mediates systemic effects of FGF21 [23, 24]. Further, KD has been shown to increase adiponectin levels in obese children and adolescents after 6 months of treatment [6]. However, given that both FGF21 and adiponectin exert beneficial effects on glucose metabolism, while a KD induces the opposite, an alteration of FGF21 effect could be expected. This would be in keeping with the observation that obese/insulin resistant high-fat diet fed mice exhibit an FGF21 resistant-state because FGF21 cannot fully initiate its action and correct insulin responsiveness when injected in these mice [25].

As KD is known to induce hepatic insulin resistance and to increase endogenous FGF21 levels [15], we investigated whether this could be associated with altered FGF21 signaling in addition to an inflammatory state. The aim of this study was therefore to understand the impact of KD on inflammation and FGF21 signaling in both the liver and WAT.

## Materials And Methods

### Animals

Male C57BL/6J mice, 8 week-old (Charles River Laboratories, L'Arbresle, France) were fed a ketogenic diet (F3666, Bio-Serv, Frenchtown, NJ, USA) or a regular chow (RC) (RM3 (E) SQC #811.181, Special Diets Services, Essex, England) for 4 weeks. The proportion of calories derived from nutrients for KD was 93.4% fat, 1.8% carbohydrate, 4.7% protein, and 7.24 kcal/g energy density; and for RC 11.5% fat, 61.6% carbohydrate, 26.9% protein, and 3.63 kcal/g energy density. Body composition was assessed by an EchoMRI-700 quantitative nuclear magnetic resonance analyzer (Echo Medical Systems, Houston, TX). Metabolic parameters and physical activity were measured using a LabMaster system (TSE Systems GmbH, Berlin, Germany) at the Small Animal Phenotyping Core Facility (University of Geneva, Geneva, Switzerland) under controlled temperature (22 +/- 1°C) and lighting (light on: 7 AM to 7 PM). All experiments were done in 6h fasted (6 AM to noon) animals, as previously described [15]. Prior to sacrifice, mice were anesthetized by pentobarbital injection (150 mg/kg). Tissues were then rapidly collected and snap-frozen in liquid nitrogen. Animal experiments were approved by the ethics committee of Geneva University School of Medicine and the Geneva State Veterinary Office.

### Plasma assays

Plasma samples were obtained as previously described [15]. Briefly, blood samples were collected by cardiac puncture in heparinized syringes and centrifuged at 12,000 rpm for 2 min. Plasma FGF21 and adiponectin were measured by ELISA kits according to the manufacturer's instructions (R & D Systems, Abingdon, UK). The adiponectin assay detected full-length adiponectin.

### Total RNA Isolation and real-time quantitative PCR analysis

Total RNA was extracted from frozen livers and abdominal WAT using the RNeasy 96 kit (Macherey-Nagel, Oensingen, Switzerland) according to the manufacturer's protocol. RNA was reverse-transcribed into cDNA with the use of Takara RT-kit (Takara Biotechnology, Saint-Germain-en-Laye, France). The abundance of transcripts was assessed by real-time PCR (ABI StepOne Plus Sequence Life Technologies, Zug, Switzerland) with a SYBR Green detection system (Roche, Rotkreuz, France). Samples were run in duplicate for the gene of interest and cyclophilin and differences were calculated using the ΔCt method. The primers used are shown in Table 1.

**Table 1:** Oligonucleotides used.

Name	Gene symbol	Forward Primers	Reverse Primers
**EGF-like module containing, mucin-like, hormone receptor-like sequence 1**	*Emr1*	CAGATACAGCAATGCCAAGCA	GATTGTGAAGGTAGCATTCACAAGTG
**CD68 antigen**	*Cd68*	ACTACATGGCGGTGGAATACAA	GATGAATTCTGCGCCATGAA
**Integrin alpha M**	*Itgam*	GATGCTTACCTGGGTTATGCTTCT	CCGAGGTGCTCCTAAAACCA
**Tumor necrosis factor alpha**	*Tnf*	CATCTTCTCAAAATTCGAGTGACAA	TGGGAGTAGACAAGGTACAACCC
**Interleukin 6**	*Il6*	GAGGATACCACTCCCAACAGACC	AAGTGCATCATCGTTGTTCATACA
**Adiponectin**	*Adipoq*	AGACCTGGCCACTTTCTCCTCATT	AGAGGAACAGGAGAGCTTGCAACA
**Fibroblast growth factor 21**	*Fgf21*	CTGGGGGTCTACCAAGCATA	TGCTATCAAGCCCTCCTTCACCAT
**NLR family, pyrin domain containing 3**	*Nlrp3*	TGCTATCAAGCCCTCCTTCACCAT	ATTTGGTCCCACACAAGCCTTTGC
**Uncoupling protein 1**	*Ucp1*	GTGAACCCGACAACTTCCGAA	TGCCAGGCAAGCTGAAACTC
**Fibroblast growth factor receptor 4**	*Fgfr4*	GCTCGGAGGTAGAGGTCTTGT	CCACGCTGACTGGTAGGAA
**Fibroblast growth factor receptor 1**	*Fgfr1*	ACTCTGCGCTGGTTGAAAAAT	GGTGGCATAGCGAACCTTGTA
**Klotho beta**	*Klb*	TGTTCTGCTGCGAGCTGTTAC	TACCGGACTCACGTACTGTTT
**Lipoprotein lipase**	*Lpl*	TTGCCCTAAGGACCCCTGAA	TTGAAGTGGCAGTTAGACACAG
**CD36 antigen**	*Cd36*	AGATGACGTGGCAAAGAACAG	CCTTGGCTAGATAACGAACTCTG
**peptidylprolyl isomerase A**	*Ppia*	GGCTCCGTCGTCTTCCTTTT	ACTCGTCCTACAGATTCATCTCC

### Western blotting

Frozen tissues (~50 mg for liver and ~100 mg for WAT) were homogenized in lysis buffer containing 150 mM NaCl, 1% (v/v) Triton X-100, 0.1% (w/v) SDS, 0.5% (w/v) Na Deoxycholate, 50 mM Tris-HCl with phosphatase and protease inhibitors (Pierce, Lausanne, Switzerland). After incubation for 15 min at 4°C, the lysate was centrifuged at 12,000 rpm for 30 min and the supernatant collected. Protein concentration was determined by BCA protein quantitative kit according to the manufacturer’s protocol (Pierce, Lausanne, Switzerland). 30 μg of total isolated proteins were loaded in each well of 4–12% SDS—PAGE gels and transferred to polyvinylidene fluoride (PVDF) membranes. Membranes were blocked with TBS containing 0.1% Tween 20 and 3% BSA, then incubated overnight with primary antibody specific for phospho-ERK1/2 (Thr202/Tyr204), ERK1/2 (dilution 1:1000, Cell signaling, Allschwil, Switzerland) and Tubulin (1:4000, Sigma-Aldrich, Buchs, Switzerland). Bound antibodies were detected using HRP-labeled secondary IgG (dilution 1:1000) and a chemiluminescence kit (Amersham, Glattbrugg, Switzerland). The blots were imaged by chemiluminescence (Supersignal West Dura substrate; Pierce, Lausanne, Switzerland) on a detection system (GE Healthcare Europe GmbH, Glattbrugg, Switzerland). Densitometric analysis of chemiluminescent signals captured on a camera was performed using the Image J software (National Institutes of Health, http://rsb.info.nih.gov/ij).

### Statistical analysis

Data are presented as means ± SEM. Statistical calculations were carried out with GraphPad Prism 6 (Prism 6; GraphPad Software, Ink, La Jolla, USA). The statistical significance of differences was determined by unpaired Student’s t-tests. A P value <0.05 was considered statistically significant.

## Results

### KD promotes weight loss through increased energy expenditure and reduced food intake

To address the systemic effect of KD, we studied 8 week-old male C57BL/6J mice fed with either KD or RC. Consistent with previous results [15], KD-fed mice exhibited a significant ~20% decrease in total body weight compared with the RC-fed group (Table 2). This decrease in body weight could be attributed to an ~37% increase in energy expenditure (Table 2) and a ~56% reduction in food intake (Table 2). However, as KD is more caloric than RC, there was no difference in caloric intake between KD and RC-fed mice (Table 2), suggesting that the decrease in body weight could be mainly attributed to higher energy expenditure in KD-fed mice. This increase in energy expenditure could not be attributed to a higher locomotor activity, as locomotor activity was even lower in KD-fed mice (Table 2). In keeping with reduced total body weight, we found that lean body mass, whether expressed in grams (Table 2) or as percentage of total body mass (Table 2), was significantly decreased in KD-fed mice. In contrast, total body fat was significantly increased by 23% in KD-fed mice compared to RC-fed mice (Table 2). Finally, body fat percentage was also increased by ~51% in KD-fed mice (Table 2).

**Table 2:** Physiological parameters and FGF21.

	RC	KD
**Body weight (g)**	26.7±0.31	21.2 ± 0.49 ***
**Lean mass (g)**	21.3 ± 0.24	15.3 ± 0.40 ***
**Lean mass (%)**	79.9 ± 0.91	72.6 ± 1.15 ***
**Fat mass (g)**	3.0 ± 0.12	3.7 ± 0.17 *
**Fat mass (%)**	11.2 ± 0.49	16.9 ± 0.78 ***
**Energy expenditure (Kcal/h/kg)**	21.1 ± 0.21	29.0 ± 0.75 ***
**Food intake (g/day/mouse)**	3.82 ± 0.07	1.68 ± 0.09 ***
**Caloric intake (Kcal/day/mouse)**	13.87 ± 0.25	12.54 ± 0.66
**Locomotor activity (photo beam breaks/day)**	35338 ± 1982	27393 ± 1780 **
**Plasma FGF21 (pg/ml)**	192.0 ± 33.20	17032 ± 4059 *
**Fold increase liver FGF21 mRNA**	1.000 ± 0.2840	13.71 ± 2.508 *
**Fold increase epididymal white adipose tissue FGF21 mRNA**	1.000 ± 0.1880 *	0.2620 ± 0.0350 *

RC, regular chow diet; KD, ketogenic diet;

*P<0.05

**P<0.01

***P<0.001.

To further characterize the metabolic profile induced by KD, we analyzed several markers involved in metabolic disorders. Because KD increased energy expenditure, we assessed the expression and plasma levels of FGF21, a central regulator of energy expenditure and lipid homeostasis. FGF21 was markedly induced as revealed by high FGF21 plasma level in KD-fed mice (Table 2). This rise in FGF21 plasma level was associated with a significant ~15 fold induction of *Fgf21* expression in the liver of KD-fed mice compared to RC-fed mice (Table 2). In addition to the liver, FGF21 is also known to be produced by the WAT (WAT). We decided to study epididymal WAT (WATe), as it is the most prominent adipose depot in mice. Hence, we determined the effect of KD on *Fgf21* expression in WATe. Interestingly, KD decreased FGF21 mRNA expression in WATe (KD vs RC: -74%, Table 2).

### KD, FGF21 signaling and inflammation

**Fig 1:**
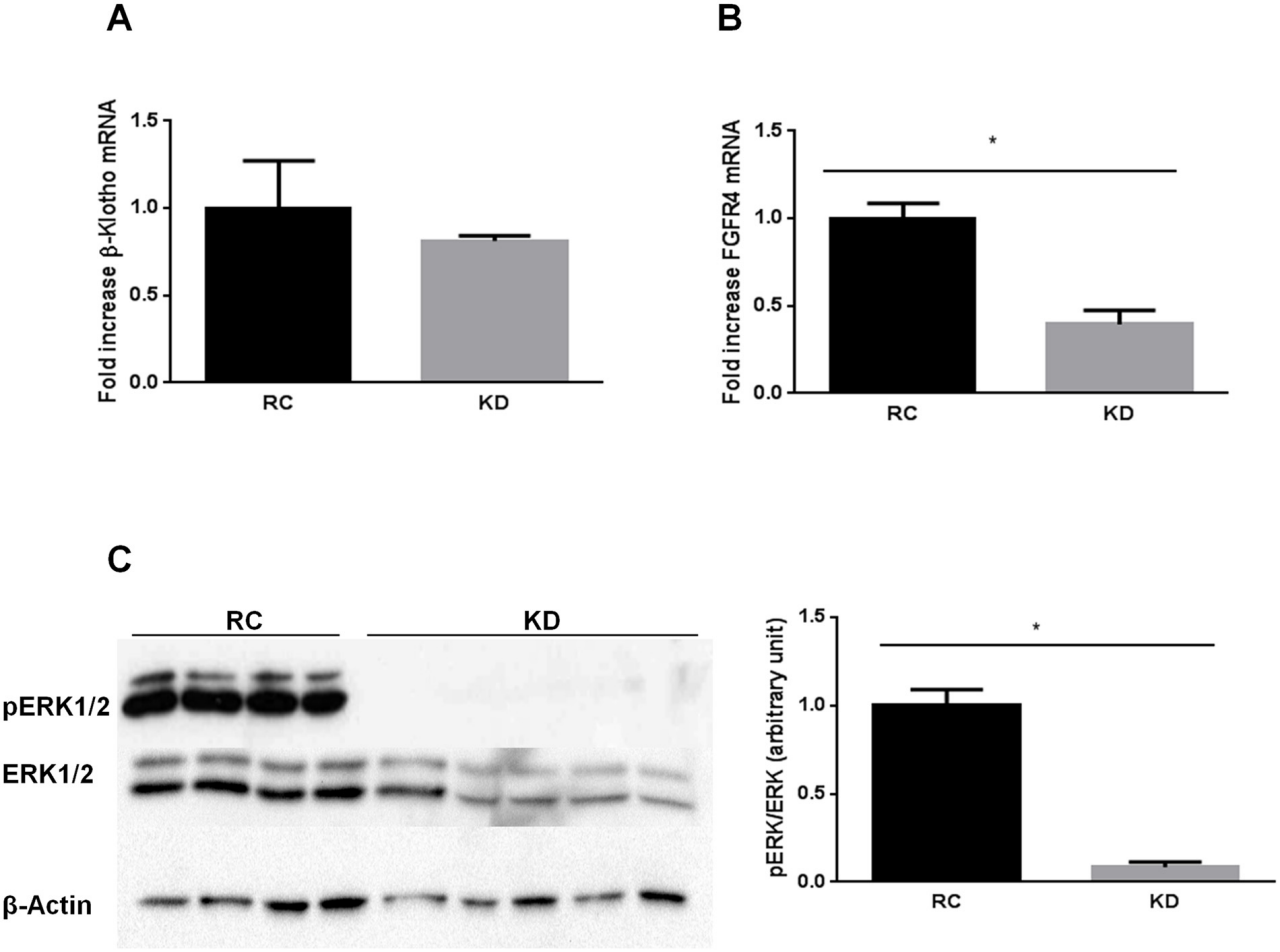
Ketogenic diet (KD) impairs FGF21 signaling in the liver. A) KD does not modify *Klb* (β-Klotho) mRNA expression, but downregulates B) *Fgfr4* mRNA expression, with a subsequent reduction of C) phospho-ERK1/2 protein levels. Data are presented as means ± SEM. * P<0.05 (n = 4–6 mice per group).

FGF21 is known to improve insulin sensitivity [26], notably in the liver. Although KD promotes FGF21 expression in the liver, such a diet has also been shown to induce hepatic insulin resistance [15]. This could be associated with an alteration in FGF21 signaling in the liver. β-klotho (*Klb*) is a required protein for FGF21 signaling [19]. We therefore determined the expression level of *Klb*, as well as the expression of FGF21 receptor 4 (*Fgfr4*), which is the main FGF21 receptor isoform in the liver. Both *Klb* and *Fgfr4* are required to activate FGF21 signaling. Surprisingly, there was no change in *Klb* expression (Fig 1A). However, KD reduced *Fgfr4* expression by 60% (Fig 1B), which may explain a further reduction in extracellular signal-regulated kinases 1/2 (ERK1/2) phosphorylation (Fig 1C), an important mediator in FGF21 signaling, although not specific. Together, these data suggest impaired FGF21 signaling in the liver of KD-fed mice. This could be due to various factors amongst which a chronic low grade inflammatory state. Indeed, it has been shown that the inflammatory cytokine TNFα impairs FGF21 action in cultured adipocytes through a downregulation of β-klotho [27].

### KD impairs FGF21 signaling in adipose tissue

**Fig 2:**
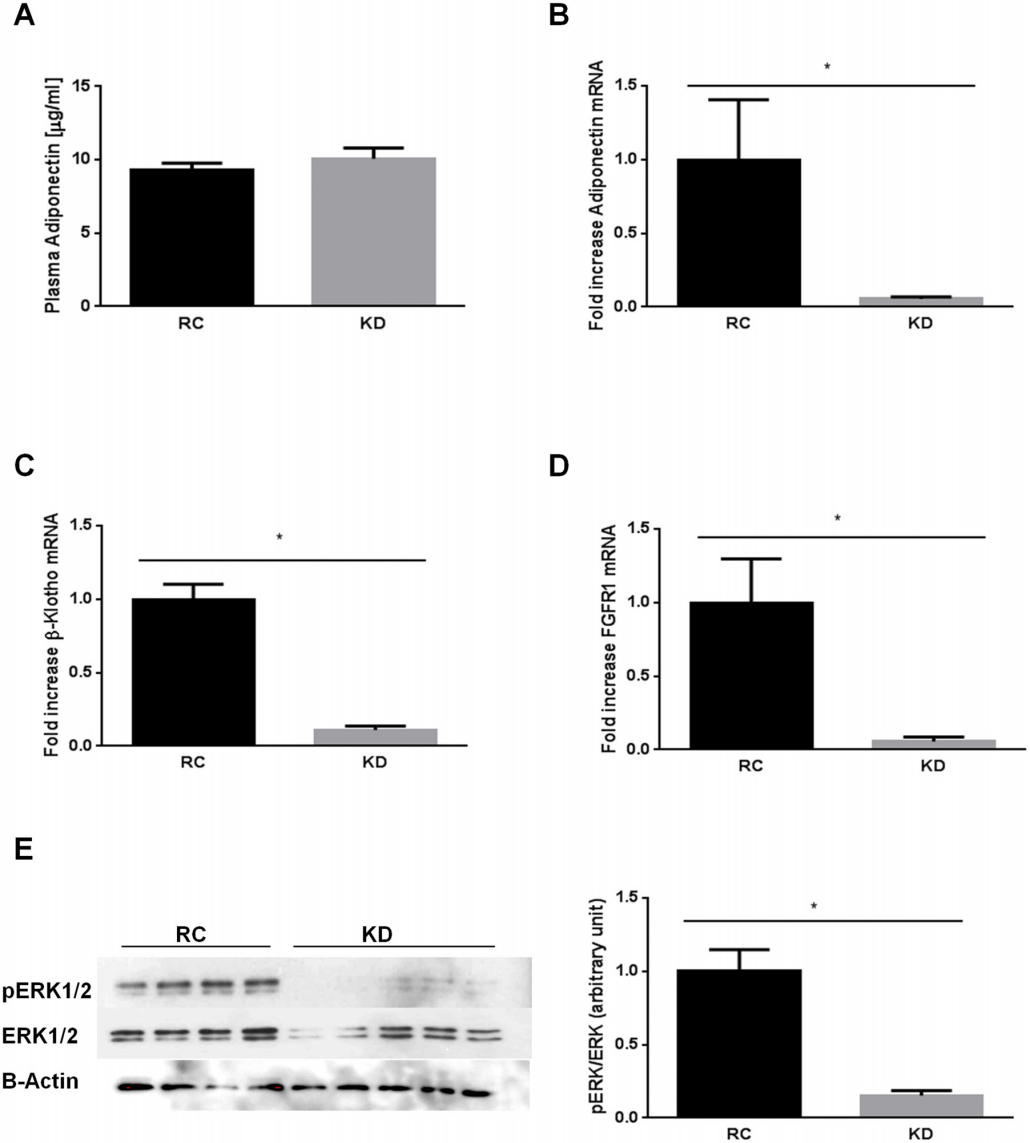
Ketogenic diet (KD) impairs FGF21 signaling in epididymal white adipose tissue. A) KD does not affect adiponectin plasma levels; KD downregulates B) *Adiponectin*, C) *Klb* (β-Klotho), D) *Fgfr1* mRNA expression, and E) phosho-ERK1/2 protein levels in white adipose tissue. Data are presented as means ± SEM. * P<0.05 (n = 4–6 mice per group).

As FGF21 metabolic effects have been shown to be mediated, at least in part, by adiponectin [23, 24], we also determined adiponectin plasma levels, as well as adiponectin expression in WATe. Surprisingly, adiponectin plasma levels in KD-fed mice were similar to those of RC-fed mice (Fig 2A). Furthermore, adiponectin mRNA expression was decreased by 95% in WAT of KD-fed mice compared to RC-fed mice (Fig 2B). Given that adiponectin is mainly produced by WAT and is targeted by FGF21, we determined whether this tissue exhibited impaired FGF21 signaling when mice were fed a KD. Altered β-klotho expression impairs FGF21 action in adipose cells [27]. We therefore evaluated *Klb* expression in WATe and found that KD highly repressed β-klotho expression by 90% compared to RC (Fig 2C). Additionally, in WATe, β-klotho is known to form a complex with the FGF21 receptor 1 (FGFR1) to transduce FGF21 signaling. We found that KD reduced *Fgfr1* mRNA expression in WAT by 95% (Fig 2D). Further, we also determined the phosphorylation level of ERK1/2, and found that KD downregulates ERK1/2 phosphorylation in WATe compared to RC-fed mice (Fig 2E).

### KD impairs FGF21 signaling in adipose tissue independently of inflammation

**Fig 3:**
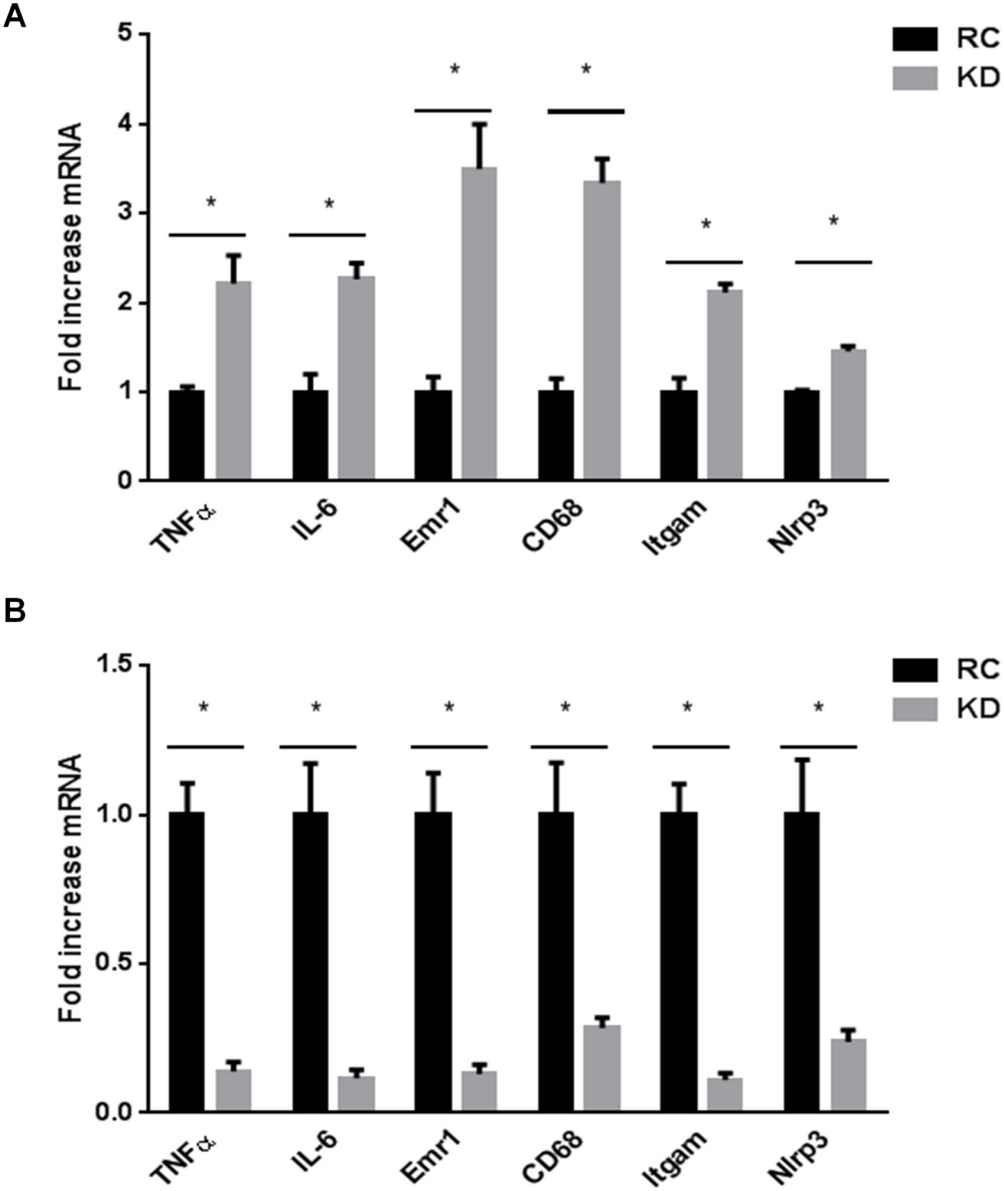
Ketogenic diet (KD) promotes inflammation in the liver while preventing it in epididymal white adipose tissue. Expression level of inflammatory markers and macrophage recruitment are A) enhanced in the liver of KD-fed mice, while B) reduced in white adipose tissue. Data are presented as means ± SEM. * P<0.05 (n = 6 mice per group).

To test the hypothesis that inflammation could mediate the alteration of FGF21 signaling, we determined the expression level of different inflammatory factors. We found that the expression of markers of inflammation (*Tnfα*, *Il-6*), macrophage accumulation (*Emr1*, *Cd68*, *Itgam*), as well as mediator of inflammasome (*Nlrp3*) was significantly increased in liver of KD-fed mice compared to RC-fed mice (Fig 3A). These results suggest that KD induces an inflammatory state that may impair hepatic FGF21 signaling. We also observed that KD-fed mice exhibit impaired FGF21 signaling in the liver and WATe. Given that alteration of FGF21 action in the liver was associated with an inflammatory state, we assessed whether a similar process could be associated with impaired FGF21 signaling in WATe. Paradoxically, we found that the expression of markers of inflammation (*Tnfα*, *Il-6*), macrophage accumulation (*Emr1*, *Cd68*, *Itgam*) as well as mediator of inflammasome (*Nlrp3*) was significantly decreased in adipose tissue of KD-fed mice compared to RC-fed mice (Fig 3B). These results suggest that, in contrast to the liver, inflammation is not associated with impaired FGF21 signaling in WATe.

### Lipid accumulation is increased in the liver but reduced in WAT of KD-fed mice

**Fig 4:**
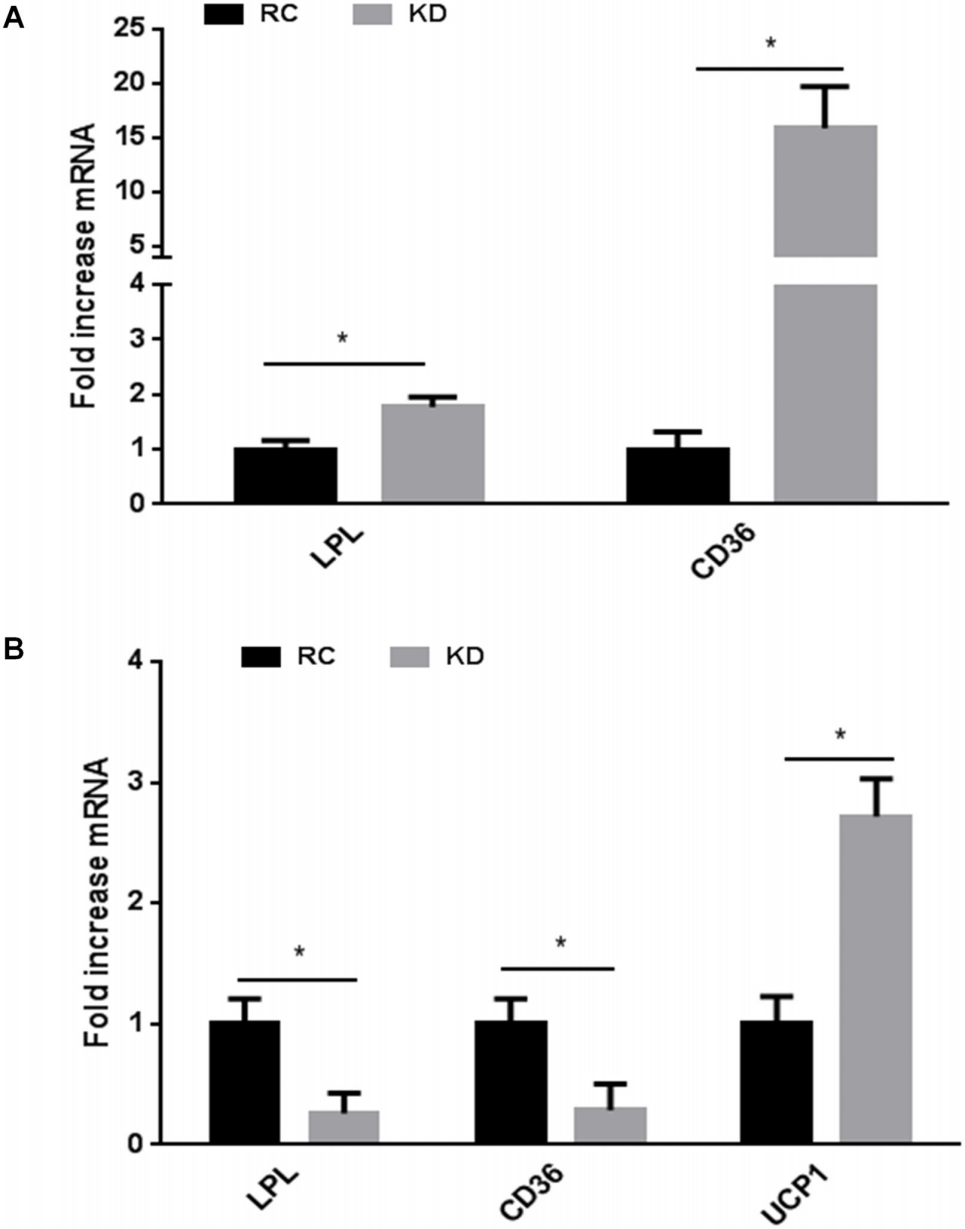
Ketogenic diet (KD) increases expression of genes involved in lipid accumulation A) in the liver while it B) reduces it in epididymal white adipose tissue. Data are presented as means ± SEM. * P<0.05 (n = 6 mice per group).

In addition to inflammation, ectopic lipid accumulation causes liver steatosis and hepatic insulin resistance. Notably, mice overexpressing lipoprotein lipase (LPL) specifically in the liver develop these alterations [28]. Because FGF21 and insulin action are likely interacting [29], we proposed that, similarly to insulin resistance, ectopic lipid accumulation could impair FGF21 signaling. To test this hypothesis, we assessed the main lipid transporters *Lpl* and *Cd36* mRNA expression in the liver and WATe of RC and KD-fed mice. We found that *Lpl* and *Cd36* were both significantly increased in the liver of KD-fed mice (Fig 4A). These data suggest an increased lipid accumulation in the liver with fatty liver as a consequence, as previously described in the same setting [15], and could explain the subsequent development of inflammation and impaired FGF21 signaling. In contrast to the liver, both *Lpl* and *Cd36* were significantly reduced in WATe of KD-fed mice (Fig 4B), suggesting a decrease in WATe lipid accumulation.

KD increases energy expenditure, and this effect is likely to be mediated by an increase in uncoupling protein-1 (UCP1) in brown adipose tissue mediated by FGF21 [15]. Moreover, FGF21 is known to induce browning of WAT [26, 30]. To explain this apparent paradox between increased energy expenditure and impaired FGF21 signaling in WATe, we determined *Ucp1* mRNA expression, which was significantly increased by KD (Fig 4B). These findings may suggest a differential action of FGF21 on different targets or an FGF21-independent UCP1 activation. As FGF21 is also known to regulate WAT lipolysis [31], it may be that fatty acids directly activate UCP1 as has been shown in brown adipose tissue [32].

## Discussion

Ketogenic diets have been reported to promote weight loss [8], but their effects on glucose metabolism are controversial [11, 15, 33]. Notably, it has been shown that KD-fed mice develop hepatic insulin resistance, while insulin responsiveness in WAT is not affected [15]. Because insulin resistance is associated with low grade inflammation [5], we report the role of KD on inflammatory processes related to metabolism in WAT and liver. Here, we report that KD promotes inflammation and lipid accumulation in the liver, while it reduces these parameters in WATe. Moreover, we show that KD impairs FGF21 signaling in both the liver and WATe.

Before further discussion, we think it is important to highlight the fact that KD has a very low protein content (4.7% of calories) and as such does not represent a physiologic state. Indeed, such a low protein content inevitably leads to catabolism, notably skeletal muscle breakdown in order to provide amino acids for gluconeogenesis, all the more that KD is by definition very low in carbohydrates. Moreover, lipids of KDs mostly contain saturated fatty acids and as such may present other adverse effects, such as cardiovascular diseases on the long term.

KD has been shown to increase triglyceride and diacylglycerol content in the liver [15]. Accumulation of such lipids may promote inflammation, which in turn could interfere with FGF21 effects by downregulating the FGF21 signaling pathway. Together, our findings suggest a link between inflammation and impaired FGF21 signaling in the liver of KD-fed mice. Previously, Garbow *et al*. showed that KD induces hepatic inflammation [14]. In keeping with these results, we found that markers of macrophage recruitment (*Emr1*, *Cd68*, *Itgam*) were significantly increased by 4 weeks of KD. However, in contrast to this study, we observed that markers of inflammation (*Tnfα*, *Il-6*) were also induced by KD. This discrepancy could be due to the duration of administration of the diet, which in our case was shorter but may have caused more acute effects in the liver. However, we compared KD-fed mice to RC-fed mice, whereas Garbow *et al*. compared KD-fed mice to mice fed a western diet (i.e. a high-fat diet) which may already by itself induce some degree of inflammation.

Additionally, Badman and coworkers found that FGF21 knockdown mice exhibited marked impairments of fatty acid oxidation when consuming KD [18]. FGF21 regulates lipid metabolism [34], suggesting that deletion of FGF21 can lead to altered lipid metabolism and ectopic accumulation of fat in the liver of mice lacking FGF21. Notably, Badman and coworkers showed that mice lacking Fgf21 gained weight and exhibited exaggerated fatty liver when fed KD [35]. In our study, we found that KD promotes weight loss and increases FGF21 plasma level and liver expression. However, KD also impaired FGF21 signaling in the liver, suggesting a reduced responsiveness to FGF21 by the liver, leading to impaired lipid metabolism and increased lipid accumulation. Therefore, our results are in line with the studies by Badman *et al*, with the exception that in our findings KD was able to promote weight loss. This discrepancy could be due to the fact that we used wild-type mice, whereas Badman *et al* used mice lacking Fgf21.

Regarding WATe, we surprisingly observed that markers of macrophage recruitment (*Emr1*, *Cd68*, *Itgam*), as well as those of inflammation (*Tnfα*, *Il-6*), were significantly reduced by KD. We therefore report a differential response regarding inflammation between liver and WATe. These results are in agreement with the study by Badman and coworkers, who found that KD significantly decreased *Il-6* mRNA expression in perigonadal adipose tissue, while it did not affect *Tnfα* expression in this same tissue [16].

KD is known to cause fat accumulation in ectopic tissues such as the liver, which in turn promotes insulin resistance [15]. We therefore investigated genes involved in lipid accumulation in both liver and WATe. *Lpl* and *Cd36* were overexpressed in the liver, and these findings are consistent with liver fat accumulation known to occur in KD-fed mice [15, 16, 36]. In contrast to what was seen in the liver, *Lpl* and *Cd36* expression were downregulated in WATe of KD-fed mice, suggesting a decrease in subsequent inflammation. Moreover, KD protects WATe from inflammation probably through a positive flow of lipids from adipose tissue to other organs, such as the liver. To support this hypothesis, we previously showed that fatty acid suppression by insulin was impaired in KD-fed mice compared to RC-fed mice, suggesting an increase in WAT lipolysis and the subsequent flow of fatty acids from WAT to ectopic tissues such as the liver [15], although the role of KD in WAT lipolysis has been questioned [17]. These results are corroborated by the differences in body composition. Indeed, KD-fed mice had a higher fat percentage after four weeks of KD and this may reflect the higher percentage of fat in the diet. However, based on the results of lipid accumulation gene expression in the liver or WATe, one can hypothesize that lipids are diverted from WAT to ectopic sites, such as the liver. This situation is similar to that occurring in mice which are devoid of Phosphatase and TENsin homolog (PTEN) in the liver, as recently described [37].

Increased plasma FGF21 levels and hepatic expression in KD-fed mice is probably secondary to hepatic fat accumulation and may represent a compensatory mechanism to counteract hepatic insulin resistance, similar to what is seen in patients with obesity, type 2 diabetes and NAFLD [38, 39]. Because FGF21 is increased by KD but cannot correct insulin resistance, we hypothesized an impaired FGF21 response in target tissues. To test this hypothesis, we evaluated part of FGF21 signaling in liver and WATe. We found that *Klb*, *Fgfr1* and ERK1/2 phosphorylation were decreased in WATe, while *Fgfr4* and ERK1/2 phosphorylation were decreased in the liver. Altogether, these data point to impaired endogenous FGF21 signaling in these tissues.

Despite impaired FGF21 signaling in WATe of KD-fed mice, we found that inflammation in this tissue was decreased. This may be due to the fact that ketone metabolism produce low levels of reactive oxygen species which are known to contribute to inflammation [40, 41]. Indeed, it has been proposed that ketone bodies exert antioxidant activity. For instance, ketone bodies block the effect of a potent oxidant compound, hydrogen peroxide [41]. By doing so, ketone bodies prevent the activation of inflammatory pathways. In contrast, inflammation observed in the liver could be due to ectopic accumulation of fat, which may generate more reactive oxygen species.

Finally, we found no change in plasma adiponectin levels between KD and RC-fed mice. As adiponectin has recently been shown to mediate FGF21 action [23, 24], these findings suggest an impaired response to endogenous FGF21 in WAT, where adiponectin is mainly produced. Surprisingly, however, we observed that adiponectin mRNA expression in WATe of KD-fed mice was lower than in RC-fed mice. This paradoxical unexplained observation could result from several mechanisms, including that other types of WAT such as the perirenal WAT may compensate to maintain adiponectin plasma levels; that KD generates a byproduct that may inhibit adiponectin production despite FGF21 elevation; or that KD could prevent FGF21 degradation.

In conclusion, KD promotes differential inflammatory and lipid accumulation responses in liver and WATe. Our findings highlight the importance of the crosstalk between these tissues. Thus, further research is warranted to better define the mechanisms leading to impaired FGF21 signaling, notably in WAT, and its link to inflammation. Indeed, KD is still largely used in the treatment of obesity, type 2 diabetes and NAFLD, but also in refractory epilepsy and as an adjuvant therapy in some cancers. Therefore, it will be important to better understand the consequences of such a diet on inflammation and FGF21 signaling and action, in order to better advise our patients facing these important and ever growing chronic diseases.
